# The role of glucagon-like peptide-1 receptor agonists (GLP1-RAs) in the management of the hypertensive patient with metabolic syndrome: a position paper from the Korean society of hypertension

**DOI:** 10.1186/s40885-024-00279-4

**Published:** 2024-09-01

**Authors:** Hae Young Lee, Seung-Hyun Ko, Sungjoon Park, Kyuho Kim, Song-Yi Kim, In-Jeong Cho, Eun Joo Cho, Hyeon Chang Kim, Jae-Hyeong Park, Sung Kee Ryu, Min Kyong Moon, Sang-Hyun Ihm, the Korean Society of Hypertension

**Affiliations:** 1 Department of Internal Medicine, Seoul National University Hospital, Seoul National University College of Medicine, Seoul, Republic of Korea; 2 Department of Internal Medicine, Division of Endocrinology and Metabolism, St. Vincent’s Hospital, College of Medicine, The Catholic University of Korea, Seoul, Republic of Korea; 3 Department of Internal Medicine, Division of Cardiology, Jeju National University Hospital, Jeju, Republic of Korea; 4 Department of Internal Medicine, Division of Cardiology, Ewha Womans University Seoul Hospital, Ewha Womans University College of Medicine, Seoul, Republic of Korea; 5 Department of Internal Medicine, Division of Cardiology, Yeouido St. Mary’s Hospital, College of Medicine, The Catholic University of Korea, Seoul, Republic of Korea; 6 Department of Preventive Medicine, Yonsei University College of Medicine, Seoul, Republic of Korea; 7 Department of Cardiology in Internal Medicine, Chungnam National University, Chungnam National University Hospital, Daejeon, Republic of Korea; 8 Wellness Healthcare Center, Ewha Womans University Seoul Hospital, Seoul, Republic of Korea; 9 Department of Internal Medicine, Division of Endocrinology & Metabolism, Seoul National University Boramae Medical Center, Seoul National University College of Medicine, Seoul, Republic of Korea; 10 Department of Internal Medicine, Division of Cardiology, Bucheon St. Mary’s Hospital, College of Medicine, The Catholic University of Korea, Seoul, Republic of Korea

**Keywords:** Metabolic syndrome, obesity, hypertension, glucagon, Like peptide, 1, glucagon, Like peptide, 1 receptor agonist

## Abstract

Obesity is the one of the most important components of metabolic syndrome. Because obesity related hypertension accounts for two thirds of essential hypertension, managing obesity and metabolic syndrome is a crucial task in the management of hypertension. However, the current non-pharmacological therapies have limitations for achieving or maintaining ideal body weight. Recently, glucagon-like peptide-1 receptor agonists (GLP1-RAs) have demonstrated excellent weight control effects, accompanied by corresponding reductions in blood pressure. GLP1-RAs have shown cardiovascular and renal protective effects in cardiovascular outcome trials both in primary and secondary prevention. In this document, the Korean Society of Hypertension intends to remark the current clinical results of GLP1-RAs and recommend the government and health-policy makers to define obesity as a disease and to establish forward-looking policies for GLP1-RA treatment for obesity treatment, including active reimbursement policies.

## Background

Metabolic syndrome (MetS) is a combination of metabolic dysfunctions mainly characterized by central obesity and insulin resistance accompanying abnormal adipose deposition and function, and the risk factors include dyslipidemia, impaired glucose tolerance, and hypertension [1]. Presence of MetS is associated with the risk of developing the cardiovascular disease (CVD) and type 2 diabetes mellitus (T2DM) [2, 3]. Obesity is one of the most important components of MetS. Obesity can cause hypertension through a variety of factors and mechanisms and obesity related hypertension accounts for 65 to 75% of essential hypertension [4–6]. Conversely, the prevalence of MetS in the hypertensive population reaches almost 60% [7]. Therefore, obesity control is the essential part not only in hypertension control but also in CVD prevention [8, 9]. However, the increase in the obese population is becoming a major obstacle in the management of hypertension from a public health perspective [10]. Overweight and obesity affect more than 50% of total population worldwide [11]. Intensive lifestyle modifications, such as diet modification, regular exercise, and alcohol moderation, have been emphasized as a treatment for obesity, but their effectiveness are limited [4, 12, 13]. In addition, there are some drugs and metabolic surgeries along with lifestyle modification to treat obesity, but they also showed limited efficacy [4, 12, 13]. However, glucagon-like peptide 1 receptor agonists (GLP1-RAs), which were recently developed as a T2DM treatment drug, has been confirmed to have a strong weight control effect as well as a CVD prevention effect [14].

The Korean Society of Hypertension (KSH) defines ‘obesity’ as a disease and strives to improve hypertension management and CVD prevention through active control, including pharmacologic therapies and metabolic surgeries along with intensive lifestyle modification. Therefore, this paper reviews the expert opinions from the KSH on the effects, side effects, and considerations of GLP1-RA in hypertensive patients with obesity. In addition, the aim of this paper is to express the views of KSH and hope that these efforts can be used as important resource for the establishment of national health policies.

## Prevalence of obesity / MetS among hypertensive patients

In recent decades, a global surge in obesity has become a significant concern, accompanied by the rising prevalence of MetS [15]. This issue is compounded by its intricate connection with hypertension, further emphasizing its need for urgent action [5]. Particular note is the pivotal role of central obesity in triggering hypertension and the heightened risk of MetS among the obese population, thereby amplifying the risks of hypertension [16]. Obesity rates vary worldwide, with Western higher-income countries showing signs of stabilization, while regions like Asia, Africa, the Middle East, and Central/South America experience a notable increase in obesity, particularly among children and adolescents, indicating a potential escalation in obesity-related health problems in the future [17, 18]. Obesity epidemic and the aging population has contributed to a rapid increase in hypertension prevalence, as highlighted by the Non-Communicable Disease Risk Factor Collaboration (NCD-RisC) study, which revealed a doubling in the global count of people with hypertension, from 648 million in 1990 to 1.278 billion in 2019 [19]. Despite these alarming statistics, the treatment and control of hypertension have shown improvement only in limited regions [20].

**Fig.1:**
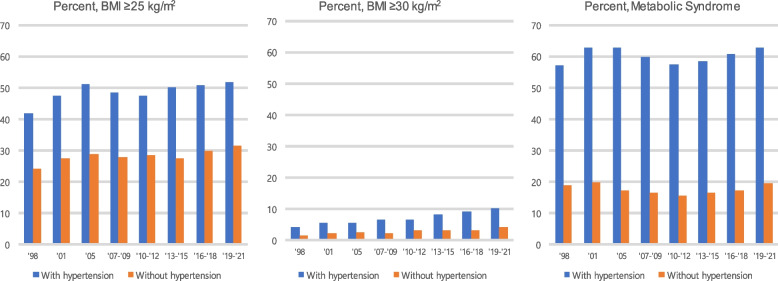
Prevalence of obesity and metabolic syndrome by the presence of hypertension among Korean adults aged 30 years or older [Data Source: KNHANES 1998–2021]. BMI, body mass index

While the Korean population exhibits a lower prevalence of obesity compared to Western populations, it has a relatively higher prevalence of MetS despite the lower obesity rates [21]. Recent trends in Korea indicate a general increase in the prevalence of both obesity and MetS [21, 22], with a notable surge in obesity observed during the COVID-19 pandemic [23]. This increase is particularly pronounced among hypertensive patients. Analysis of the Korean National Health and Nutrition Examination Survey (KNHANES) data reveals that among adults aged 30 and above, the prevalence of obesity (defined as body mass index (BMI) ≥ 25 kg/m^2^) increased from 42% in 1998 to 52% in 2019–2021 among those with hypertension, while it increased from 24 to 31% among those without hypertension during the same period. Notably, the prevalence of severe obesity (BMI ≥ 30 kg/m^2^) increased from 4.3% to 10.4% among hypertensive individuals and from 1.7% to 4.3% among those without hypertension during the same period. The prevalence of MetS in adults aged ≥ 30 years with hypertension is strikingly high at 63%, compared to 20% among those without hypertension. This indicates that approximately two-thirds of hypertensive individuals have MetS (Fig. 1). These findings underscore the need for comprehensive management, addressing not only blood pressure (BP) control but also obesity and obesity-related metabolic complications among the majority of hypertensive patients [24]. Furthermore, it suggests that a significant portion of individuals requiring GLP1-RA treatment are hypertensive patients.

### Current treatment strategy for obesity

Lifestyle modification including reducing energy intake, physical activity and behavioral therapy are the basic step of obesity treatment. A low-calorie diet that reduces energy intake by 500–1,000 kcal per day can make weight loss without negative health effect [25]. A low-carbohydrate diet (defined as daily carbohydrate intake of more than 130 g and less than 45% of total energy) led to greater initial weight loss than traditionally recommended low-fat diets and was associated with a greater improvement in CVD risk factors [26, 27]. And a systematic review and network meta-analysis that compared 14 dietary macronutrient patterns showed that most macronutrient diets resulted in modest weight loss over 6 months, but weight reduction and improvements in cardio-metabolic factors diminished after 12 months [28]. Adequate protein intake is extremely important in calorie restriction for preventing muscle mass loss regardless of diet [29].

Exercise is very important for improving lifestyle habits for weight loss. A systematic review and network meta-analysis showed that exercise led to a significant reduction of weight, fat and visceral fat. The effect of aerobic and high-intensity interval training with same energy expenditure was equal. Resistance training reduced lean mass loss during weight loss [30]. Clinical practice guideline for obesity by Korean Society for the Study of Obesity (KSSO) recommends aerobic exercise for at least 150 min per week, 3 to 5 times a week, and resistance exercise using large muscle groups 2 to 3 times per week for weight loss. Furthermore, increasing aerobic exercise to 250 to 300 min per week is suggested for more meaningful weight loss. Behavioral therapy to improve lifestyle such as reducing food intake and increasing physical activity is also recommended for weight loss and maintenance. Abstinence from alcohol and smoking also is recommended if weight loss of 2.5% is not achieved within 1 month of obesity therapy [31].

KSSO recommends that pharmacotherapy should be considered when intensive lifestyle modifications fail to achieve a weight reduction in obese patients with a BMI ≥ 25 kg/m^2^ and that the pharmacotherapy be changed or discontinued if weight loss is not greater than 5% within 3 months of pharmacotherapy [32]. Currently four types of obesity treatments have been approved for long-term administration in Korea: orlistat, naltrexone-bupropion, liraglutide, and phentermine-topiramate [33]. In patients with hypertension, orlistat, phentermine/topiramate ER, and liraglutide can be used and heart rate should be monitored in patients receiving phentermine/topiramate ER, and liraglutide. In case of patients with established atherosclerotic CVD, orlistat and liraglutide are recommended [34].

Indications of bariatric/metabolic surgery in Korean adults are i) with a BMI of 35 kg/m^2^ or more, or ii) a BMI of 30 kg/m^2^ or more with obesity-related comorbidities, who have failed to lose weight with non-surgical treatment and iii) with T2DM with a BMI of 27.5 kg/m^2^ or more and a blood glucose level that is not properly controlled with non-surgical treatment. Before performing bariatric/metabolic surgery, it is necessary for comprehensive evaluation about the patient's physical condition as well as psychosocial history and appropriate evaluation of nutritional status is required.[31].

### Action mechanism and pharmacologic profile of GLP1-RAs

The connection between the pancreas, the gut, and incretin hormones was first described in the early part of the twentieth century [35]. The first incretin to be extracted from gut mucosa was gastric inhibitory polypeptide [36]. Glucagon-like peptide-1 (GLP1), a proglucagon cleavage product made by intestinal L cells, was identified in the early 1980s [37].

The promise of GLP1 as a therapeutic target in T2DM was appreciated when it was demonstrated that it contributes up to 70% of insulin production in response to nutrition intake [38]. Although natural GLP1 at pharmaceutical levels could restore the insulin secretory response in patients with T2DM, the utilization of this peptide for therapeutic purposes has been constrained by its short half-life [39]. To overcome the short half-life of endogenous GLP1 and to achieve therapeutic advantages, two approaches are being investigated; dipeptidyl peptidase-4 (DPP4) inhibitors, which prevent the breakdown of native GLP1, and the synthesis of GLP1 analogs with prolonged action [40]. Several strategies have been used to extend the biological effect of GLP1-RAs [41]. They include amino acid changes or variants to confer resistance to cleavage by DPP4 (exenatide and lixisenatide), dilution with chemical adjuncts such as zinc to delay absorption from subcutaneous tissue (taspoglutide), covalent conjugation with large molecules such as albumin or IgG to retard renal elimination (albiglutide, dulaglutide, and semaglutide), the attachment of fatty acid side chains to confer noncovalent albumin binding (liraglutide), and coupling to biodegradable polymer microspheres to confer protracted release from the subcutaneous tissue (exenatide extended-release) [41]. Presently, taspoglutide and albiglutide have been discontinued by the developer. Taspoglutide was discontinued due to instances of serious hypersensitivity reactions and gastrointestinal side effects. Albiglutide was discontinued due to limited prescribing of the drug, not due to any safety concerns in 2018. Overcoming the limitations of injectable drugs, the oral formulation of semaglutide is now licensed for use. Oral semaglutide is combined with sodium N-[8-(2-hydroxybenzoyl) amino]- caprylate (SNAC), which allows entry of semaglutide in the circulation with a bioavailability of around 1% by functioning as an absorption enhancer [42].

One of the primary actions of GLP1-RAs is to enhance insulin secretion from the pancreatic β-cells in a glucose-dependent manner [43, 44]. Unlike other insulin secretagogues that may stimulate insulin release regardless of blood glucose levels, GLP1-RAs only enhance insulin secretion when blood glucose levels are elevated. This reduces the risk of hypoglycemia, a common side effect associated with some diabetes treatments. By increasing insulin levels when needed, GLP1-RAs help lower blood glucose levels effectively. GLP1-RAs also suppress the secretion of glucagon, a hormone that increases blood glucose levels by promoting glucose production in the liver. By inhibiting glucagon release, GLP1-RAs reduce hepatic glucose output, further contributing to the reduction in blood glucose levels. This suppression occurs in a glucose-dependent manner as well, ensuring that the risk of hypoglycemia is minimized. Another significant effect of GLP1-RAs is the slowing of gastric emptying. This means that after eating, the stomach takes longer to empty its contents into the small intestine. This delay helps moderate the rise in blood glucose levels postprandially (after meals) by slowing the rate at which glucose is absorbed into the bloodstream. Moreover, this effect contributes to a feeling of fullness (satiety), which can help reduce food intake and support weight loss efforts in patients with type 2 diabetes. Some actions of GLP1-RAs, most notably the inhibition of gastric emptying [41]. GLP1-RAs have been shown to have a central effect on appetite regulation, acting on the brain to increase feelings of fullness and reduce hunger. This can lead to a decrease in calorie intake and contribute to weight loss, an important aspect of managing type 2 diabetes, as obesity is a major risk factor for the development and progression of the disease.

GLP1-RAs are primarily eliminated by the kidney. Dosing adjustments of GLP1-RAs are unnecessary due to hepatic or mild renal impairment. However, patients with moderate renal impairment should avoid weekly exenatide, and dose escalations should be carefully evaluated in patients using twice-daily exenatide. Similarly, lixisenatide usage needs to be monitored carefully in the patient population with renal impairment [45]. Dulaglutide, liraglutide, and semaglutide are approved for use down to the estimated glomerular filtration rate (eGFR) of 15 mL/min/1.73 m^2^. Table 1 is a summary of currently available GLP1-RAs, their basic characteristics, and indications according to renal impairment.

**Table 1:** Characteristics of GLP1-RA

Drug	Structural class	Half-life	Administration	Renal function in eGFR^a^
**Short-acting**				
Exenatide	Exendin-4 derivative	~ 2.4 h	Subcu. twice daily	Avoid if eGFR < 30
Lixisenatide	Exendin-4 derivative	3–4 h	Subcu. once daily	Avoid if eGFR < 30, caution if eGFR 30–50
**Long-acting**				
Dulaglutide	Modified human GLP1	~ 90 h	Subcu. once weekly	Can use down to eGFR 15
Exenatide extended-release	Exendin-4 derivative		Subcu. once weekly	Avoid if eGFR < 30
Liraglutide	Modified human GLP1	11 ~ 13 h	Subcu. once daily	Can use down to eGFR 15
Semaglutide	Modified human GLP1	~ 7 days	Subcu. once weekly, oral once daily	Can use down to eGFR 15

^a^eGFR in mL/min/1.73 m^2^

*eGFR* estimated glomerular filtration rate, *GLP1-RA* glucagon-like peptide-1 receptor agonists, subcu., subcutaneous

### Body weight reducing effects with GLP1-RAs

GLP1 primarily modulates energy balance by promoting insulin secretion and additionally, it influences feeding behavior by impacting various neural circuits associated with appetite [46, 47]. GLP1 and GLP1-RAs decrease appetite and food intake by enhancing satiety and abdominal fullness with both intracerebroventricular and peripheral administration [48]. Extended treatment of GLP1-RAs at doses slightly above the standard T2DM treatment dose results in weight loss. While the reduction in HbA1c levels plateaus at relatively lower doses, higher doses may remain more effective for achieving weight loss [49, 50].

The observation that certain GLP1-RA, like albiglutide, exhibit relatively modest impact on body weight, while others, such as semaglutide, demonstrate more substantial effects despite similar glucose-lowering efficacy, has prompted interest in understanding the underlying mechanism of action. Recent data about the effects of semaglutide (and liraglutide) on obesity induced by diet in rodents suggest that the impact of systemically administered GLP1-RA on appetite, satiety, calorie intake, and body weight involves the arcuate nucleus in the hypothalamus, the area postrema, and the nucleus tractus solitarius.[51, 52] Interestingly, GLP1-RA can impact food choices by promoting the selection of healthier, less energy-dense foods in human studies [53, 54].

### Prevention of new onset DM with GLP1-RAs

In adults with overweight or obesity at high risk of T2DM, care strategy should focus on weight loss to minimize the progression of hyperglycemia and associated comorbidities [55]. There are strong and consistent evidences that obesity management through intensive lifestyle modification can delay the progression from prediabetes to T2DM [56, 57]. A loss of 5–10% of body weight leads to improved lipid profile, BP, glycemic control status, and reduced incidence of T2DM, with greater benefits achieved with sustained weight loss of > 10% [58]. Based on several clinical trials, the US Food and Drug Administration (FDA) has approved two subcutaneous GLP1-RAs, liraglutide (3 mg once daily) and semaglutide (2.4 mg once weekly), as weight loss medications for long-term use in individuals with BMI ≥ 30 kg/m^2^ or BMI ≥ 27 kg/m^2^ with at least one weight-associated comorbidity, regardless of T2DM status [59]. These GLP1-RAs have been shown significant weight loss benefits. Liraglutide achieves a mean weight loss of 4–7 kg, and ≥ 50% of treated subjects achieve ≥ 5% weight loss. Semaglutide has a greater impact with a mean weight loss of 9–16 kg, and ≥ 50% of treated subjects achieve ≥ 10–15% weight loss. In the SCALE (Satiety and Clinical Adiposity e Liraglutide Evidence) obesity and prediabetes trial (*n* = 3,731), the mean weight loss after 56 weeks of liraglutide treatment (3.0 mg once daily) was 8.4 kg vs. 2.8 kg and a third of participants loss > 10% of their overall weight [60]. Among them, 2,254 prediabetic subjects were evaluated after a 3-year-long treatment. At the end of follow-up, conversion rate to T2DM was significantly lower in liraglutide group (6% vs 2%, placebo vs. liraglutide group, respectively), and the time to onset of T2DM over the study period was 2.7 times longer with liraglutide [61]. In the STEP (Semaglutide Treatment Effect in People with Obesity) 1 trial, semaglutide treatment (2.4 mg once weekly) for 68 weeks (*n* = 1,961) showed that reversion of prediabetes occurred in 84.1% of patients compared to 47.8% of controls [62].

A systematic review and meta‐analysis including eight eligible studies evaluated the beneficial effect of GLP‐1RA on prediabetes with overweight/obesity [63]. In this study, more individuals in GLP1-RAs group regressed from prediabetes to normoglycemia than subjects in the placebo group (OR = 4.56, 95% CI 3.58–5.80); fewer individuals in GLP1-RAs group were diagnosed with T2DM than those in the placebo group (OR = 0.31, 95% CI 0.12–0.81). Results of this study revealed that GLP1-RA treatment in prediabetes significantly lowered weight, fasting glucose, waist circumference, and systolic BP. In the meta-analysis including a total of 31 randomized controlled trials (*n* = 22,948), the mean differences (95% CI) of the pooled GLP1-RA-induced change in the HbA1c level was -0.78% (-0.97—-0.60%) in the random-effects model and -0.45% (-0.47—-0.44%) in the fixed-effect model. The pooled body weight reduction was -4.05 kg (-5.02—-3.09 kg) in the random-effects model and -2.04 kg (-2.16—-1.92 kg) in the fixed-effect model [64].

In summary, GLP1-RAs could be a promising regimen for prediabetes with overweight/obesity, particularly in terms of delaying the progression from pre-diabetes to T2DM. The effect of GLP‐1RA on the prediabetes with normal weight or the length of the treatment period needs to be refined further.

### BP lowering effects with GLP1-RAs

Hypertension and diabetes often coexist, affecting approximately 60% of people aged 30 years or older with diabetes in Korea. However, among them, only half of the people achieved the target goal for BP [65]. Moreover, the combination of these two conditions increases the risk of CVD, making BP-lowering crucial for the reduction of CVD in hypertensive patients with diabetes. GLP1-RAs consistently lower BP in clinical and experimental studies.

Asmar et al. reported that acute intravenous administration of GLP-1 leads to significantly increased systolic BP and heart rate [66]. Whereas, chronic administration of GLP1-RAs has consistently reduced BP in several cardiovascular outcome trials (Table 2) [67–75]. In the ELIXA study (The Evaluation of Lixisenatide in Acute Coronary), the addition of lixisenatide to usual care did not significantly reduce the major cardiovascular outcomes, however, it did show a significant decrease in systolic BP (mean -0.8 mm Hg) and an increased heart rate (+ 0.4 beats per minute) compared to the placebo group [67]. In the LEADER study (the Liraglutide Effect and Action in Diabetes: Evaluation of Cardiovascular Outcome), the first study of positive cardiovascular outcomes of GLP1-RA, systolic BP was lower (-1.2 mm Hg), but diastolic BP was higher (+ 0.6 mm Hg), and heart rate (+ 3.0 beats per minute) was increased in the liraglutide group compared to the placebo group [69]. Moreover, the SUSTAIN-6 trial (Evaluate Cardiovascular and Other Long-term Outcomes with Semaglutide in Subjects with Type 2 Diabetes) showed dose-dependent systolic BP decrease and an increase in heart rate in semaglutide group [68]. These findings were similar to the PIONEER 6 study (Peptide Innovation for Early Diabetes Treatment) with oral semaglutide [74]. The BP-lowering effect of GLP1-RAs was similar between the short-acting and long-acting formulations. The result Harmony outcomes (Albiglutide and cardiovascular outcomes in patients with type 2 diabetes and cardiovascular disease) with albiglutide, a long-acting formulation of GLP1-RA, slightly reduced the mean systolic BP in the albiglutide group compared with the placebo group, and BP difference was similar between 8 and 16 months (-0.65 and -0.67 mm Hg at 8 months and 16 months) [71]. The REWIND (Researching Cardiovascular Events with a Weekly Incretin in Diabetes) study with a long-acting dulaglutide showed similar findings with systolic BP decrease and heart rate increase [72]. In the meta-analysis, compared with placebo, and other antidiabetic treatments including insulin, and sulfonylureas, GLP1-RAs decreased systolic BP (range from -1.84 mm Hg to 4.60 mm Hg) compared with the placebo group, but diastolic BP was only significantly reduced in exenatide use (-1.08 mm Hg) [76].

**Table 2:** Change difference of body weight and blood pressure (BP) in major cardiovascular outcomes trials of GLP1-RAs

Study acronym	GLP1-RA	Follow up duration	Population	Baseline BMI	Body weight change (kg)	Systolic BP change
ELIXA [67]	Lixisenatide	25 months	T2DM with CVD	30.2	-0.7 kg	-0.8 mmHg
LEADER [69]	Liraglutide	3.8 years	T2DM with high CVD risk	32.5	-2.3 kg	-1.2 mmHg
SUSTAIN-6 [68]	Semaglutide	2 years	T2DM	33	–2.9 kg (0.5 mg)—–4.3 kg (1.0 mg)	-1.3 mmHg (0.5 mg) -2.6 mmHg (1.0 mg)
EXSCEL [70]	Exenatide	3.2 years	T2 DM with / without CVD	31.8	-1.27 kg	-1.57 mmHg
Harmony Outcomes [71]	Albiglutide	1.6 years	T2DM with CVD	32.3	–0.83 kg	–0.67 mmHg
REWIND [72]	Dulaglutide	5.4 years	T2 DM with ≥ 50 years with a previous CVD or ≥ 60 years ≥ 2 CVD risks	32.3	-1.46 kg	-1.70 mmHg
PIONEER6 [73]	Semaglutide Oral	15.9 months	≥ 50 years with CVD or ≥ 60 years with CVD risk factors	32.3	–3.4 kg	-2.6 mmHg
STEP-HFpEF [74]	Semaglutide	1 year	HFpEF with BMI ≥ 30 kg/m2	37	-14 kg	-2.9 mmHg
SELECT [75]	Semaglutide	34.2 months	Non-DM ≥ 45 years with CVD and BMI > 27 g/m2	33.4	-9.1 kg	-3.82 mmHg

All trials were multicenter, double-blind, randomized placebo-controlled trial. T2DM, type 2 diabetes mellitus, *CVD* cardiovascular disease

*DMI* body mass index, *BP* blood pressure, *CVD* cardiovascular disease, *DM* diabetes mellitus, *HFpEF* heart failure with preserved ejection fraction, *GLP1-RA* glucagon-like peptide-1 receptor agonists

There are several mechanisms postulated regarding BP reduction with GLP1-RAs [77, 78]. Weight reduction was positively associated with systolic and diastolic BP reduction in a meta-analysis [79]. However, this BP-lowering effect occurred in the early period, within 2–4 weeks of drug administration before significant weight loss, suggesting that BP reduction might be independently associated with weight loss [77, 80, 81]. These effects became plateau within 8–12 weeks and were maintained up to 2 years [77]. GLP1 receptor has been found to be expressed in endothelial cells and vascular smooth muscle cells in several organs including the heart, brain, kidney, and blood vessels [77, 78]. In animal studies, GLP1-RA treatment increased endothelial nitric oxide synthase [82], led to direct vascular relaxation [83], decreased vascular remodeling,[84] and reduced intercellular adhesion molecule expression,[82] mitigating endothelial dysfunction, vascular inflammation, and arterial stiffness [78]. GLP1-RA treatment enhanced the secretion of atrial natriuretic peptide on atrial cardiomyocytes in a mouse model, promoting natriuresis and smooth muscle relaxation [85]. Also, the administration of GLP1 or GLP1-RAs induced dose-dependent diuresis and natriuresis, linked to increased glomerular filtration rate in rodents [86] and obese men.[87] GLP1-RAs suppress sympathetic activation on the central (hypothalamus and brainstem) [88] and peripheral nervous system (carotid body) in animal studies [89]. Consequently, GLP1-RAs have favorable effects on BP-lowering by mitigating endothelial dysfunction and vascular contraction, preventing vascular remodeling, promoting diuresis and natriuresis, and suppressing sympathetic activation.

### Ancillary effect on vascular function with GLP1-RAs

GLP1-RAs improve endothelial function in several ways in addition to their glucose-lowering effects. Increased nitric oxide (NO) production is the first mechanism. In human vascular endothelial cells, liraglutide increased NO production by stimulating phosphorylation of endothelial nitric oxide synthase, the enzyme that produces NO, in a 5' AMP-activated protein kinase-dependent manner [90]. The second mechanism is reducing oxidative stress. GLP1-RAs reduced reactive oxygen species and the expression of vascular cell adhesion molecule-1 mRNA in endothelial cells after exposure to advanced glycation end products [91]. In addition, GLP1-RAs reduce oxidative stress by increasing the production of antioxidants such as glutathione and NO. The third mechanism is the improvement of mitochondrial function. GLP1 RAs improve mitochondrial function by increasing mitochondrial biogenesis and reducing mitochondrial apoptosis [92]. It recovers mitochondrial membrane potential, oxygen consumption and myeloperoxidase levels. The final mechanism is the reduction of inflammation. GLP1-RAs have anti-inflammatory effects through a variety of mechanisms, including the reduction of pro-inflammatory cytokine production and the increase of anti-inflammatory cytokine production. GLP1-RAs inhibit the formation of macrophage foam cells [93]. Treatment with liraglutide increased NO production and reduced tumor necrosis factor α -induced nuclear factor kappa B activation [90]. Several GLP1-RAs have been shown to be effective in the reduction of systemic inflammation, as measured by C-reactive protein levels [94].

Beyond their effects on glucose metabolism and weight, GLP1-RAs also influence lipid metabolism [95, 96]. These agents have been shown to modulate lipid synthesis and secretion, contributing to improved lipid profiles in patients with T2DM. The mechanisms behind these effects are multifaceted. GLP1-RAs can reduce hepatic lipogenesis, the process by which the liver synthesizes fatty acids and triglycerides. Additionally, they may enhance the clearance of lipids from the bloodstream, through mechanisms that include increased lipoprotein lipase activity, leading to a reduction in circulating triglycerides. Furthermore, GLP1-RAs have been observed to impact the secretion of very low-density lipoprotein (VLDL) particles by the liver, which are a major carrier of triglycerides.

GLP1-RAs have beneficial effects on the vasculature in two ways, directly and indirectly. GLP1-RAs can directly relax vascular smooth muscle cells, thereby reducing vascular stiffness. Liraglutide, independent of its glucose-lowering effect, may inhibit angiotensin II-induced vascular smooth muscle cell proliferation by activating AMP-activated protein kinase signaling and inducing cell cycle arrest, thereby delaying the progression of atherosclerosis [97]. Also, GLP1-RAs can produce NO that relaxes blood vessels and improves blood flow and have favorable effects on endothelial function indicators, such as the reactive hyperemia index and flow-mediated dilatation, these can attenuate vascular stiffness [98, 99]. Indirectly, GLP1-RA induced positive vascular effects may be associated with improvements in glucose and lipid metabolism, weight reduction, and BP lowering effects. GLP1-RAs can improve metabolic profiles and reduce vascular adipose tissue-derived inflammation, subsequently [100]. Additionally, they have a favorable effect on BP, improving vascular stiffness.

The potential impact of GLP1-RAs on CV risk factors extends to thrombosis and platelet aggregation [101]. Emerging evidence suggests that GLP1-RAs may exert protective effects against thrombosis by influencing the function of platelets and the coagulation cascade. These agents have been shown to reduce platelet activation and aggregation, mechanisms that are crucial in the formation of thrombi and the development of cardiovascular events such as myocardial infarction and stroke. The anti-thrombotic effects of GLP1-RAs are thought to be mediated through both direct and indirect pathways. Directly, GLP1-RAs may influence platelet function through GLP-1 receptors expressed on platelets themselves. Indirectly, the reduction in systemic inflammation, improvement in endothelial function, and amelioration of atherosclerotic changes associated with GLP-1RA therapy may contribute to their anti-thrombotic effects.

### Target organ protection by GLP1-RAs

Through several clinical studies, the cardiovascular protective effects of GLP1-RAs have been demonstrated. In the LEADER study, SUSTAIN-6 study, Harmony Outcomes study, and REWIND study, liraglutide, semaglutide, albiglutide, and dulaglutide showed a significant reduction in major adverse cardiac events (death from cardiovascular causes, nonfatal myocardial infarction, nonfatal stroke) compared to the placebo group, respectively [68, 69, 71, 72]. On the other hand, in the ELIXA study, EXSCEL study, and PIONEER 6 study, exenatide, extended-release exenatide, and oral semaglutide groups demonstrated non-inferiority regarding cardiovascular safety compared to the placebo, respectively[67, 70, 73]. When meta-analyzing these seven randomized controlled trials, GLP1-RAs were found to reduce major CVD events by 12% (hazard ratio [HR], 0.88; 95% CI, 0.82–0.94), CVD mortality by 12% (HR, 0.88; 95% CI, 0.81–0.96), and stroke risk by 16% (HR, 0.84; 95% CI, 0.76–0.93) [102]. Very recently, semaglutide reduced the risk of major cardiovascular events by 20% compared with placebo in non-diabetic patients who are overweight or obese and with pre-existing CVD [75].

GLP1-RAs have also shown potential benefits in heart failure. Meta-analysis revealed promising trends regarding heart failure outcomes in terms of reduced rate of heart failure-related events [103]. The effect of Semaglutide 2.4 mg Once Weekly on Function and Symptoms in Subjects with Obesity-related Heart Failure with Preserved Ejection Fraction (STEP-HFpEF) trial reported that semaglutide led to greater weight loss, heart failure related symptom improvement and to significant difference in 6-min walk distance when compared to placebo in patients with preserved left ventricular ejection fraction ≥ 45% and obesity (BMI ≥ 30 kg/m^2^) [74].

GLP1-RAs, particularly longer-acting formulations, reduced stroke events in T2DM [104]. The effects of GLP1-RAs on stroke subtypes gave discordant results in placebo-controlled outcome trials. An exploratory analysis in the REWIND trial indicated that dulaglutide might reduce the incidence but not severity of ischemic stroke (3.2% on dulaglutide versus 4.1% on placebo, corresponding to a 24% risk reduction with dulaglutide versus placebo) [105]. However, no effects were seen on hemorrhagic stroke. In contrast, a post hoc analysis of SUSTAIN 6 and PIONEER 6 showed that semaglutide reduced the risk of any stroke by 32% compared with placebo, with no difference between stroke subtypes [106].

The renal effects of GLP1-RAs have garnered significant attention in recent years, reflecting a broader understanding of their benefits beyond glycemic control in patients with T2DM [107, 108]. The renal benefits of GLP1-RAs are mediated through several mechanisms, which include improving hemodynamics, reducing inflammation, and attenuating oxidative stress in the kidneys. These agents have been shown to reduce glomerular hyperfiltration, a condition commonly seen in the early stages of diabetic nephropathy, by improving the tubuloglomerular feedback. This results in a reduction of intraglomerular pressure, thereby slowing the progression of kidney damage. Additionally, GLP1-RAs can decrease albuminuria, which is an early marker of diabetic nephropathy and a predictor of renal and cardiovascular outcomes. The reduction in albuminuria with GLP1-RAs is believed to be independent of their glucose-lowering effects and may result from direct anti-inflammatory and antifibrotic effects on the kidneys. These agents are also thought to improve renal outcomes by reducing systemic and renal inflammation, as evidenced by decreases in markers of inflammation such as C-reactive protein and interleukin-6. Several large cardiovascular outcome trials (CVOTs) have highlighted the renal benefits of GLP1-RAs. The LEADER trial, which investigated liraglutide, demonstrated a significant reduction in the risk of new-onset persistent macroalbuminuria, although there were no significant differences in the rates of doubling of serum creatinine, the need for renal replacement therapy, or death due to renal disease. Similarly, the SUSTAIN-6 trial with semaglutide showed a reduction in new or worsening nephropathy. These findings are complemented by real-world studies and meta-analyses that further support the renal protective effects of GLP1-RAs. In a meta-analysis, GLP1-RAs were shown to reduce the risk of renal composite outcomes (occurrence of sustained macroalbuminuria, doubling of serum creatinine, glomerular filtration rate decrease of 30% or 40%, need for renal replacement therapy, or death due to renal disease) by 17% [102]. And GLP1-RAs are reported to have positive effects on non-alcoholic fatty liver disease (NAFLD) [109]. In patients with NAFLD, liraglutide reduced liver and visceral fat, improving liver histology and function [110].

### Adverse effects of GLP1-RAs

The most common side effects of GLP1-RAs are gastrointestinal symptoms, including nausea, vomiting, and diarrhea, which are the main reasons for drug discontinuation [111]. The incidence of adverse reactions in the digestive system varies depending on the type and dose of the GLP1-RAs and the type of concomitant hypoglycemic agents. Nausea mostly disappears after a few weeks of administration and can be minimized by starting with a low dose and gradually increasing the dose. While nausea and vomiting are more common with short-acting agents, side effects such as itching or nodules at the injection site appear more frequently with long-acting agents and usually disappear in 3 to 4 weeks [112]. The safety and tolerability of GLP1-RAs with respect to renal function are generally favorable. However, like any therapeutic agents, they are associated with potential side effects, including gastrointestinal symptoms, which are the most common. There is also a theoretical risk of acute kidney injury, primarily through volume depletion due to nausea or vomiting, but this risk is considered low and manageable with appropriate patient monitoring and hydration.

GLP1-RA therapy has been associated with an increased risk of gallbladder and biliary tract diseases, including cholelithiasis and cholecystitis. In a meta-analysis including 76 studies, GLP1-RA therapy significantly increased the relative risk of the composite outcome of gallbladder or biliary tract disease [113]. In particular, the risk increased with the use of GLP1-RAs for weight loss, high doses, and long-term treatment. Post-marketing surveillance reports also reported an increased risk of acute cholecystitis due to GLP1-RA treatment.[114] Animal model studies have suggested that GLP1-RAs may cause pancreatitis and exocrine dysplasia [115], however, in large randomized controlled trials, GLP1-RAs did not increase the risk of pancreatitis or pancreatic cancer. Currently, the US FDA and the European Medicines Agency have concluded that there is no direct possibility with GLP1-RAs [116].

Although the risk of hypoglycemia is very low due to its glucose-dependent mechanism of action, the risk of hypoglycemia may increase when used in combination with medications that can cause hypoglycemia [117]. Antibodies to GLP1-RAs may develop. Generally, the titer of antibodies decreases over time and does not affect glycemic control.

In rodent animal models, GLP1-RA increases thyroid parafollicular cell (C-cell) proliferation and tumorigenesis [118]. This is because not only do rodent C-cells express more GLP1 receptors than humans, but there is also a higher incidence of thyroid C-cell carcinoma. There are no data in humans, but it should not be used in patients with a past or family history of medullary thyroid cancer or multiple endocrine neoplasia type 2 [119]. Angioedema and anaphylaxis have been rarely reported with GLP1-RAs, including semaglutide, liraglutide, dulaglutide, exenatide, and lixisenatide [120].

### Position of GLP1-RAs in the clinical guidelines

GLP1-RAs have increasingly become integral to the management of T2DM and, more recently, have been recognized for their cardiovascular benefits. Their inclusion in clinical guidelines reflects their efficacy in improving glycemic control, promoting weight loss, and reducing cardiovascular risk. This discussion focuses on the role of GLP1-RAs in current clinical guidelines, particularly those related to diabetes and cardiovascular disease management.

In the management of T2DM, GLP1-RAs are recommended as part of a comprehensive treatment strategy that may include lifestyle modifications, metformin (considered the first-line treatment in most guidelines), and other glucose-lowering medications. The American Diabetes Association (ADA) and the European Association for the Study of Diabetes (EASD), in their consensus report, highlight the use of GLP1-RAs, especially in patients with T2DM who have established atherosclerotic CVD, heart failure, or chronic kidney disease, given their proven benefit in reducing major adverse CV events (MACE) [121]. The guidelines prioritize drugs with proven cardiovascular benefits for patients with T2DM and established CVD or indicators of high risk. GLP1-RAs, along with the sodium-glucose cotransporter-2 (SGLT2) inhibitors, are preferred in such cases due to their ability to address both glycemic control and cardiovascular risk factors effectively. Emerging evidence of the cardiovascular benefits of GLP1-RAs has led to their inclusion in guidelines beyond diabetes management. For instance, the 2019 European Society of Cardiology (ESC) guidelines for the management of T2DM, pre-diabetes, and CVD developed in collaboration with the EASD recognize the role of GLP1-RAs in reducing the risk of MACE in patients with T2DM and CVD [122]. These guidelines recommend considering these agents as part of the treatment regimen for such patients, reflecting a shift towards a more CV protective approach in managing T2DM.

### Economical concern of GLP1-RAs

The economic burden of obesity on healthcare systems is substantial, making effective and cost-efficient treatments essential. The aggregate medical cost of obesity in the U.S. was 260 billion USD in 2016 (1.5% of the gross domestic product), equating to 20% of all health care expenditures [123]. Globally, it is estimated that obesity-related complications will cost 1.2 trillion USD by 2025, and a 5% weight loss in obese patients can improve their health and reduce the incidence of obesity-related complications [124]. The economic consideration of using GLP1-RAs in obesity care can be examined from multiple angles. The primary economic advantage of GLP1-RAs in obesity care lies in their potential to reduce obesity-related healthcare costs. By facilitating weight loss and improving metabolic health,these medications can mitigate the need for treatments and hospitalizations relatedto obesity-related conditions. This leads to lower direct medical costs for healthcare systems.

Several studies have assessed the cost-effectiveness of using GLP1-RAs in obesity care. These studies consider several factors such as medication costs, the degree of weight loss achieved, and the potential reduction in obesity-related healthcare expenditures. The cost-effectiveness of anti-obesity medications were estimated in obese population model of 78.2% females with a mean age of 45 years and BMI of 37.1 kg/m^2^ for women and 36.8 kg/m^2^ for men [125]. Of five anti-obesity medications, tirzepatide, semaglutide, liraglutide, phentermine plus topiramate, and naltrexone plus bupropion, of which monthly medication costs were estimated as approximately 739, 1023, 1023, 151, and 230 USD, respectively, phentermine plus topiramate was considered as the most cost-effective treatment, mainly because of its low price and similar effectiveness. To achieve a cost-effectiveness ratio of 150,000 USD, the price of tirzepatide would need to be reduced more than 38% to become cost-effective need. In a cost-effectiveness analysis of four GLP-1RA (liraglutide 1.8 mg once daily, semaglutide 1.0 mg weekly, dulaglutide 1.5 mg weekly, or exenatide 10 μg twice daily) compared with no-treatment group. Their monthly medication costs were estimated as 921.9, 827.7, 813.6, and 729.6 USD, respectively. Only semaglutide provided a cost-effective strategy based on a willing-to-pay threshold of $195,000/quality-adjusted life years (QALYs) with an incremental cost-effectiveness ratios (ICER) of $135,467/QALY, owing to superior efficacy of BMI reduction.[126] In case of semaglutide, semaglutide 2.4 mg in the treatment of adult patients with obesity (ie, BMI ≥ 30 kg/m^2^) and adult patients who are overweight (ie, BMI 27–29.9 kg/m^2^) with 1 or more weight-related comorbidities was estimated to be cost-effective compared with no treatment, diet and exercise alone, and all other branded anti-obesity medications (liraglutide 3 mg, phentermine-topiramate, and naltrexone-bupropion) under a willing-to-pay threshold of 150,000 USD per QALY gained over a 30-year time horizon [127]. In this study, the cost for semaglutide 2.4 mg was estimated as 17,597.48 USD annually, and was estimated to be particularly cost-effective in the subgroup of obese patients with BMI ≥ 40 kg/m^2^ without T2DM. However, in less risk population of adolescents ages 15 years and older with BMI ≥ 37 kg/m^2^, top-dose phentermine and topiramate might be the preferred strategy with an ICER of 56,876 USD per QALY gained vs lifestyle counseling. Semaglutide was projected to yield the most QALYs, but with an unfavorable ICER of 1.1 million USD per QALY gained, which would need to be reduced more than 85% to become cost-effective.[128]. Their monthly medication costs were estimated as 191 and 1,295 USD, respectively. Even semaglutide was considered as less cost-effective compared with sleeve gastrectomy [129]. For semaglutide to be cost-effective when compared with sleeve gastrectomy, it would have to cost less than 1,879 USD (class III), 1,204 USD (class II) or 297 USD (class I) annually.

It is undeniable that GLP1-RAs for weight loss is expensive, far exceeding to cost-effective range. However, this limitation can be substantially reduced if GLP1-RAs are reimbursed by health insurance system for at least high-risk obese patients. However, the insurance coverage of anti-obesity medications is very limited. A study analyzing 136 marketplace health insurance plans showed that just 11% had some coverage for any kind of anti-obesity medications in the US [130]. An important consideration in the anti-obesity pharmacotherapy with GLP1-RA is the health equity. Even in diabetic patients who were commercially insured, Asian, Black, and Hispanic individuals had lower use of GLP1-RA, while higher household income was independently associated with higher use [131]. However, for expanding access for diabetic patients, obesity aside, in low-income and middle-income countries, GLP1-RA would require price reductions by approximately 98% (208 USD annually), to meet a common cost-effectiveness threshold of achieving incremental costs per incremental DALY averted less than three times the GDP per capita over sulfonylurea therapy alone [132].

### Practical tips of GLP1-RAs in obesity care

The use of GLP1-RAs in clinical practice involves several practical considerations, including patient selection, choice of agent, and strategies for initiation and up-titration. The selection of patients for GLP1-RA therapy should be individualized, considering factors such as the patient's cardiovascular risk profile, need for weight loss, glycemic targets, and potential side effects. GLP1-RAs are particularly beneficial for patients with T2DM who have a high risk of CVD or established CVD, given the cardiovascular benefits demonstrated in several CVOTs. Also, overweight or obese patients could benefit from weight reduction.

The choice among GLP1-RAs should be based on factors such as the agent's efficacy, safety profile, dosing frequency, patient preference, and cost. Some GLP1-RAs are administered via subcutaneous injection daily, while others are available as weekly formulations, which may be more convenient for patients and improve adherence. The efficacy in terms of glycemic control and weight loss, as well as cardiovascular benefits, varies slightly among agents, so choosing an agent should also consider the individual patient's health goals and risk factors. Initiating GLP1-RA therapy involves starting at a lower dose to minimize gastrointestinal side effects, such as nausea and vomiting, which are the most common adverse effects associated with these agents. A gradual up-titration of the dose is recommended until the therapeutic dose is reached or the maximum tolerated dose is identified. This approach helps improve tolerability and patient adherence to therapy. Patient education is crucial during this phase to set realistic expectations regarding potential side effects and the importance of adherence to the titration schedule. Monitoring the patient's response to GLP1-RA therapy is essential for ensuring optimal outcomes. This includes regular assessments of glycemic control, weight, blood pressure, and potential side effects. Adjustments to the therapy may be necessary based on the patient's response and tolerability. In some cases, combining GLP1-RAs with other antidiabetic medications may be considered to achieve glycemic targets.

### Gut-CV connection with GLP1-RAs

The gut-CV connection highlights the intricate interplay between the gastrointestinal system and CV health, an area of growing research interest. One significant aspect of this connection is the role of the gut microbiome, which comprises trillions of microorganisms residing in the gastrointestinal tract. These microorganisms have a profound impact on metabolic, immunological, and physiological processes, including those related to cardiovascular health. GLP1-RAs, a class of medications primarily used in the management of T2DM, have been shown to offer CV benefits, and emerging evidence suggests that their effects may be partly mediated through the gut microbiome [133]. Research suggests that the gut microbiota can influence the efficacy of GLP1-RAs in several ways. Firstly, the composition of the gut microbiota affects the metabolism and bioavailability of these drugs, potentially influencing their therapeutic effects. Secondly, GLP1-RAs have been shown to alter the composition of the gut microbiota, promoting the growth of beneficial bacterial species that have been associated with improved metabolic outcomes and reduced cardiovascular risk. Moreover, the gut microbiome influences the production of short-chain fatty acids (SCFAs) through the fermentation of dietary fibers. SCFAs have been shown to have several beneficial effects on cardiovascular health, including anti-inflammatory properties, blood pressure regulation, and improvement of lipid metabolism. GLP1-RAs may enhance the production of SCFAs by altering the gut microbiota composition, thereby contributing to their CV benefits.

### Ongoing CV outcome trials of GLP1 agonists

The ongoing CVOTs of GLP1-RAs are pivotal in understanding the impact of these therapies on cardiovascular outcomes in patients with T2DM and a history of or high risk for CVD. Table 3 is a summary of the ongoing CVOTs withe GLP1-RAs.

**Table 3:** Ongoing cardiovascular outcomes trials of GLP1-RAs

Agent	Company	Development stage	Indication	ClinicalTrials.gov ID
**GLP1/glucagon dual agonists**				
Cotadutide	AstraZeneca	Phase III	NASH	NCT05364931 (active, not recruiting)
Survodutide	Boehringer Ingelheim	Phase III	Obesity	NCT06066515 (recruiting)
Efinopegdutide	MSD	Phase II	NASH	NCT05877547 (recruiting)
Mazdutide	Eli Lilly	Phase III	Obesity	NCT05607680 (active, not recruiting)
DA-1726	NeuroBo Pharmaceuticals	Phase I	Obesity	NCT06252220 (not yet recruiting)
**GIP/GLP1 dual agonists**				
Tirzepatide	Eli Lilly	Phase II	NASH	NCT04166773 (active, not recruiting)
NN9709	Novo Nordisk	Discontinued	Obesity, T2D	NCT02205528 (completed)
**GIP/GLP1/glucagon tri-agonists**				
HM15211	Hanmi Pharmaceutical	Phase II	NASH	NCT04505436 (recruiting)
Retatrutide	Eli Lilly	Phase III	Obesity	NCT05882045 (recruiting)
NN9423	Novo Nordisk	Discontinued	Obesity, T2D	NCT03661879 (completed)
**GLP1R agonists**				
Efpeglenatide	Hanmi Pharmaceutical	Phase III	T2D	NCT03353350 (completed) NCT03496298 (terminated)
		Phase II	NASH	NCT04505436 (recruiting)
Danuglipron	Pfizer	Phase II	Obesity	NCT04707313 (completed)
		Phase II	T2D	NCT03985293 (completed)
Orforglipron	Eli Lilly	Phase III	T2D	NCT05872620 (recruiting)
			Obesity	NCT05869903 (recruiting)
Lotiglipron	Pfizer	withdrawn		

## Summary and conclusion

1. Managing obesity and metabolic syndrome is a crucial task in the management of hypertension. However, the current non-pharmacological therapies have limitations.
2. GLP1-RAs demonstrate excellent weight control effects, accompanied by corresponding reductions in BP.
3. GLP1-RAs have shown cardiovascular and renal protective effects in cardiovascular outcome trials both in primary and secondary prevention.
4. The Korean Society of Hypertension defines obesity as a disease and aims to improve hypertension control rates and prevent CVDs through active management, including drug therapy. In this regard, there is a positive evaluation of the weight loss and BP control effects of GLP1-RAs.
5. High costs and gastrointestinal side effects impose restrictions on usage. Future research should focus on cost-effectiveness studies regarding the management of CVDs in comparison to drug prices. Additionally, it is urged that the government classifies obesity as a disease and establishes active reimbursement policies for control medications.

## Data Availability

Not applicable.

## Abbreviations

ADA: American Diabetes Association
BMI: Body mass index
CVD: Cardiovascular disease
eGFR: Estimated glomerular filtration rate
EASD: European Association for the Study of Diabetes
ESC: European Society of Cardiology
FDA: US Food and Drug Administration
GLP1: Glucagon-like peptide-1
GLP1-RAs: Glucagon-like peptide-1 receptor agonists
KSH: Korean Society of Hypertension
KSSO: Korean Society for the Study of Obesity
MetS: Metabolic syndrome
NAFLD: Non-alcoholic fatty liver disease
NO: Nitric oxide
QALYs: Quality-adjusted life years
SGLT2: Sodium-glucose cotransporter-2
SNAC: Sodium N-[8-(2-hydroxybenzoyl) amino]-caprylate
T2DM: Type 2 diabetes mellitus
